# A method to identify prescription drug targets for health technology reassessment

**DOI:** 10.1017/S026646232510322X

**Published:** 2025-11-28

**Authors:** Mark Hofmeister, Michael R. Law, Cheryl A. Sadowski, Rosmin Esmail, Fiona Clement

**Affiliations:** 1 Department of Community Health Sciences, University of Calgary, Calgary, AB, Canada; 2 Centre for Health Services and Policy Research, The University of British Columbia, Vancouver, BC, Canada; 3 Faculty of Pharmacy and Pharmaceutical Sciences, University of Alberta, Edmonton, AB, Canada; 4 Acute Care Alberta, Calgary, AB, Canada

**Keywords:** technology assessment, biomedical, prescription drugs, drug utilization

## Abstract

**Introduction:**

The simultaneous existence of low-value health care and underutilization of high-value care are global problems. Health technology reassessment (HTR) aims to optimize the value for money of technologies already in use within health care. Identifying candidate interventions for HTR remains challenging. Therefore, we tested a novel method to identify candidate outpatient prescription drugs for HTR through practice variation.

**Methods:**

We used administrative data for all publicly funded outpatient prescriptions dispensed to persons aged 65 or older in Alberta in 2023. Through quantitative comparison of funnel plots for Anatomic Therapeutic Chemical (ATC) classes at the fourth level stratified by prescriber specialty, variation in prescription dispensation rates between prescribers was used to estimate three outcomes: the number of prescribers affected, the number of patients affected, and the potential budgetary impact. We ranked combinations of ATC class and prescriber specialty in descending order for each outcome, with use above and below the mean considered separately.

**Results:**

We analyzed data on 17.5 million dispensations, encompassing more than 8,000 prescribers and approximately 600,000 patients. The top ATC class–prescriber specialty combinations for each outcome showed high similarity above and below control limits while exhibiting minimal overlap between outcomes.

**Conclusions:**

Our method successfully identified ATC class–prescriber specialty combinations with marked variation in use, for potential advancement through the HTR process. Depending on the perspective of those undertaking HTR of prescription drugs, different outcomes may be useful in technology prioritization. To make the ATC class–prescriber specialty combinations actionable, future efforts should focus on exploring the patients affected.

## Introduction

In the context of finite resources and competing priorities, optimizing the value for money of healthcare spending is essential to maximize patient outcomes and ensure the sustainability of healthcare delivery (1–3). Overuse of low-value care and the simultaneous underuse of high-value care comprise suboptimal care, affecting patients, the health system, and society. At the patient level, preventable diseases and complications increase patient suffering, morbidity, and mortality (2). At the health system level, poorly managed conditions lead to increased use of other resources such as emergency services, hospitals, and physician time (1). At the societal level, suboptimal care leads to decreased economic output, exacerbation of inequity due to vulnerable populations being disproportionately affected, and increased social costs due to reliance on social services and support systems (4).

Suboptimal care is increasingly recognized globally among countries from all income brackets (2;5). For example, unnecessary antibiotic prescriptions to treat upper respiratory infections have been identified in high-income countries such as the United States, the United Kingdom, and Sweden and in low- and middle-income countries such as Brazil, China, India, Russia, and South Africa (2). Studies from the United States and Australia estimated that only 55 percent of recommended care was provided to patients (5). In 2013, high-value care was not provided to more than 400 million people globally, including a lack of antenatal care for pregnant women, a lack of treatments for human immunodeficiency virus (HIV)–positive adults and children, and a failure to treat adults with new cases of tuberculosis (5).

One process to address suboptimal care is health technology reassessment (HTR). HTR is the structured evaluation of a healthcare technology’s clinical, social, ethical, and economic impacts relative to other alternatives (6). HTR focuses on technologies already in use in the healthcare system, seeking to optimize system sustainability and patient outcomes simultaneously (6). HTR does not require a previous health technology assessment conducted prior to adoption. The only requirement for HTR is that a healthcare technology is currently in use; it does not require a previous health technology assessment. The conceptual model for HTR outlines three phases: identification and prioritization of candidate technologies; evidence synthesis and policy development; and policy implementation, monitoring, and evaluation (6). Foundational components of meaningful engagement and ongoing knowledge exchange and utilization support the HTR process (6). This work focuses specifically on the first phase of the HTR process: identifying and prioritizing candidate technologies.

Guided by the conceptual model, nondrug technologies for advancement through the HTR process have been successfully identified (6;7). Soril et al. (7) assembled an expert advisory group to guide the process for the Health Technology Assessment Committee in British Columbia, whose decision making remit excludes drugs. Key process attributes defined by the expert advisory group were data-driven, routine and replicable, actionable, enabling collaboration, and offering a high return on investment (7). To identify nondrug candidate technologies, lists of low-value care, such as “Choosing Wisely” or “Do Not Do” recommendations, were compiled (7). Over 1,300 care recommendations were coded to match British Columbia administrative data assets, focusing on patient diagnoses and technology usage (7). Recommendations were ranked based on the estimated budgetary impact of current use across the province (7). Ultimately, this approach produced a list of nine nondrug candidate technologies for reassessment in British Columbia, each with a budget impact exceeding 1 million Canadian dollars per year (7). Four hundred seventy-four care recommendations for prescription drugs were not considered (7). Despite the large number of care recommendations for low-value use of drug technologies, there is a paucity of HTR efforts addressing prescription drug utilization (7;8).

Prescription drugs constitute an opportune area of service to apply the HTR process. First, spending on prescription drugs is substantial. Low-value care has been estimated to make up nearly 30 percent of healthcare spending (2;9). The global pharmaceutical market was estimated to exceed $1.5 trillion by 2023 (10). If the same prevalence of low-value care were present in the global pharmaceutical market, roughly $450 billion could have been spent differently on pharmaceuticals to achieve greater health improvements and/or not spent to achieve substantial cost reductions.

Second, formulary management tools are familiar and practical tools to optimize patient health outcomes and access to prescription drugs while managing costs (11). Active formulary management can optimize health outcomes by prioritizing the use of drugs with strong evidence of benefit, enhancing access through increased use of generic substitution, managing costs through price negotiations, and influencing demand through listing decisions, copayments, or coinsurance. Formulary management tools impact large groups of prescribers and patients simultaneously, making them an effective lever to change prescription drug usage.

Despite how attractive prescription drug targets are for HTR, identifying candidate technologies remains challenging (12). Prescription drugs causing harm can be readily identified through adverse event registers (12;13). Other technology identification methods for HTR include evidence surveillance, nomination by experts, mobilization of low-value care lists, or assessment of variation (7;12). These other methods can identify changes in evidence of effectiveness, new comparators, changes in indications for use or target population, or even obsolescence. However, challenges remain when searching for technologies that fail to offer benefits, or benefits are too small to be worth the added costs (12;14). Drug technology identification methods for HTR are needed that consider all prescription drugs simultaneously. This would allow decision makers to compare the return on investment between drugs, supporting evidence-based decision making.

This work tests a novel method to identify candidate outpatient prescription drugs for the HTR process within a publicly funded formulary. Guided by the process attributes of data-driven, routine and replicable, actionable, enabling collaboration, and offering a high return on investment (7), we leverage administrative data to identify prescription drug targets for HTR that may be amenable to alterations in use with formulary management tools.

## Methods

### Theoretical context

Like the work of Hollingworth et al. (15), this analysis builds on Wennberg’s “professional uncertainty hypothesis,” which asserted that high variation reflects clinical uncertainty about appropriate use. Further, significant and persistent variations suggest multifactorial causes, including spurious variations in care, and differences in financial incentives, clinical needs, and/or patient preferences (15). Identifying high variation may help policy makers prioritize areas where HTR is needed and where resource reallocation may be appropriate (15). The present work seeks to identify prescription drugs subject to large variations in use between prescribers, where HTR is needed, and for which resource reallocation may be optimized through formulary management tools.

### Data sources

Our period-prevalence drug utilization study included community dispensations of prescription drugs in 2023. We used geographically defined administrative data that captured all outpatient dispensations for those over age 65 in Alberta (the population with public prescription drug funding) from the province’s Pharmaceutical Information Network (PIN) database. The following prescription dispensation information was captured at each dispensation event: patient identifier; date of service; drug, strength, and dosage according to drug identification number; days supplied; number of units dispensed; and list price (16;17).

PIN data were deterministically linked with the Practitioner Claims database to obtain the following characteristics of prescribers: practice size above age 65 (number of unique patient identifiers with billing claims from a provider), mean age of patients in prescriber practice (calculated with patient age at first billing claim in 2023), proportion of patients that are female in prescriber practice (calculated with patient sex at first billing claim in 2023), and clinical specialty (estimated with most frequent specialty of billing claims used in 2023).

### Analysis

Prescriptions dispensed from the Alberta Drug Benefit List (ADBL) (17) were grouped using the World Health Organization’s Anatomic Therapeutic Chemical (ATC) classifications at the fourth level (18). The ATC system is widely used among drug utilization studies and groups drugs according to the organ/system affected and their therapeutic, pharmacological, and chemical properties (19;20). The ATC system has five levels, from level 1, representing the anatomical main group, to level 5, which represents unique chemical substances (19). Within the fourth level of ATC classification, drugs are often used to treat the same indication but may not be entirely substitutable (21). MacDonald and Potvin (21) argue that variation at the fourth level of ATC classification is of greater clinical relevance for high-level comparisons than variation in unique chemical substances.

**Figure 1.**
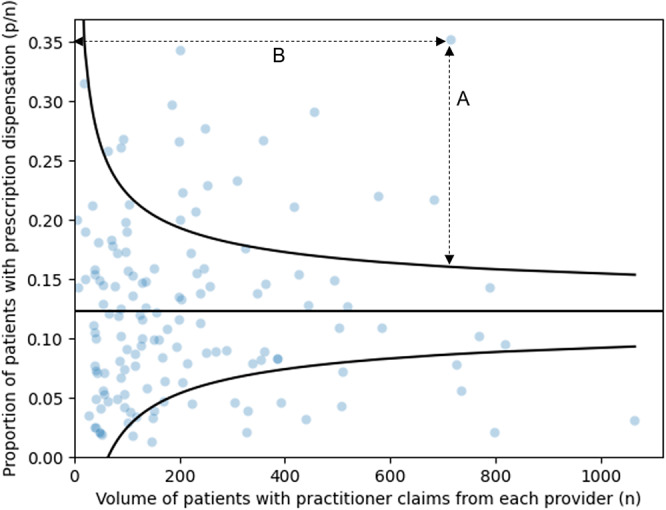
Funnel plot for C03CA: Sulfonamides, plain, showing estimation of patients that might be affected and potential budgetary impact. Funnel plot is for prescribers primarily billing under the cardiology specialty.

Spiegelhalter funnel plots were generated for each combination of ATC class at the fourth level and prescriber specialty (22). Analysis was stratified by prescriber specialty to reduce heterogeneity. For example, indications for upadacitinib include both Crohn’s disease and rheumatoid arthritis (23). Prescribers from different medical specialties may use the same drugs and ATC classes to treat different indications. Four key components of funnel plots are (i) an indicator *Y*, in this case, the proportion of patients with a dispensation for a given ATC class; (ii) a target for the indicator *θ_0_*, which is the horizontal line representing the provincial mean proportion of patients with a dispensation; (iii) a precision parameter, which for a proportion is Var(Y|*θ_0_*) = θ0 (1 − θ0)/*n*, where *n* is the practice size or number of unique patient identifiers for which a prescriber has billed; and (iv) control limits, corresponding to three standard deviations from the target *θ_0_* (Figure 1).

Each data point on funnel plots (Figure 1) represents a unique prescriber identifier, with the proportion of patients with a dispensation calculated as the number of unique patient identifiers with at least one dispensation divided by the practice size above age 65 for the prescriber. Funnel plots included prescribers with at least one dispensation of the ATC class in question. Curved control limits indicate precision in the provincial mean proportion of patients with a dispensation as a function of practice size and are set at three standard deviations from the mean. Shewhart (24) suggests that control limits set at three standard deviations are sufficient to differentiate common-cause variability from special-cause variability. Mohammed et al. (25) argue that Shewhart’s approach remains valid in healthcare settings.

For each funnel plot, we estimated three outcomes that a decision maker might use to identify prescription drug candidates for HTR offering high return on investment: number of prescribers that might be affected, number of patients that might be affected, and potential budgetary impact in 2023 Canadian dollars. These three outcomes were selected for their ease of interpretation, comparability between technologies, and immediate occurrence of the outcome alongside dispensation events.

The number of prescribers that might be affected was the number of data points outside control limits at three standard deviations. The number of patients outside control limits was calculated as the product of the vertical distance between data points and the control limit (Figure 1, line A) and practice size (line B). To estimate the potential budgetary impact, the number of patients that might be affected was multiplied by the estimated mean price for each ATC class in 2023. The price of each dispensation was calculated as the listed unit price from the ADBL as of July 1, 2023 (17), multiplied by the number of doses dispensed. Within therapeutically equivalent drug products, such as angiotensin-converting enzyme (ACE) inhibitors or proton pump inhibitors, the ADBL defines the maximum allowable cost paid for a specific drug product (17). Where available, the maximum allowable cost was used in price calculations. The mean price per patient in 2023 for each ATC class at the fourth level was the mean of dispensation prices across all included patients with a dispensation, stratified by prescriber specialty. Data on dispensing fees were not available and are excluded from this analysis.

Each of the three outcomes considered was summed separately for prescribers above and below control limits. Funnel plots for each ATC class–prescriber specialty combination were ranked in descending order for each outcome to identify the top ten ATC classes stratified by prescriber specialty. Analysis was conducted in the Python programming language (26). Research ethics was approved by the Conjoint Health Research Ethics Board (ID: REB22-0737_REN1) at the University of Calgary.

## Results

We identified 8,136 unique prescribers in our data set (Table 1). Nearly half of the prescribers used “general practice” billing codes most frequently, followed by “optometry” and “internal medicine.” There were 40 different specialties of billing codes present in our data. The mean age of patients in prescriber practices was nearly 75 years, with mostly female patients in their practices. Seventeen point five million dispensations were captured for 624,547 patients across 315 unique ATC classes at the fourth level. The most numerous dispensations were for hydroxymethylglutaryl-coenzyme A reductase (HMG-CoA) reductase inhibitors, proton pump inhibitors, antidepressants, thyroid hormones, and ACE inhibitors.

**Table 1.** Prescriber characteristics

Characteristics	Value	
Number of unique prescriber identifiers (n)	8,136	
Top three specialties of prescribers (n)	General practice = 4,240 Optometry = 638 Internal medicine = 469 Others = 2,789	
Mean practice size above age 65 for prescribers (n)	152.09 ± 13.52	
Mean age of patients in practice (years)	74.86 ± 2.12	
Mean proportion of female patients in practice	0.54	
Unique ATC classes at the level of therapeutic exchangeability (n at the fourth level)	315	
Total number of dispensations (n)	17,474,568	
Number of unique patient identifiers (n)	624,547	
Dispensations of most common ATC classifications at the fourth level (n)	**ATC class**	**Number of dispensations (n)**
	C10AA: HMG-CoA reductase inhibitors	1,536,597
	A02BC: Proton pump inhibitors	1,023,358
	N06AX: Other antidepressants	847,237
	H03AA: Thyroid hormones	802,247
	C09AA: ACE inhibitors, plain	741,047

Abbreviations: ATC, anatomic therapeutic chemical; ACE, angiotensin-converting enzyme.

The sum of potential budgetary impact above control limits across all ATC class–prescriber specialty combinations was nearly $85 million, ranging from $32,308,733 to zero. Values above control limits represent potential cost reductions if all prescribers above control limits were brought down to control limits. There were 3,740 ATC class–prescriber specialty combinations with zero potential budgetary impact above control limits. The sum of potential budgetary impact below control limits across prescriber specialties was smaller, at less than $20 million. Below control limits, the potential budgetary impact ranged from $6,573,131 to zero. Values below control limits represent potential cost increases if all prescribers below control limits were brought up to control limits. There were 4,868 ATC class–prescriber specialty combinations with zero potential budgetary impact below control limits.

**Figure 2.**
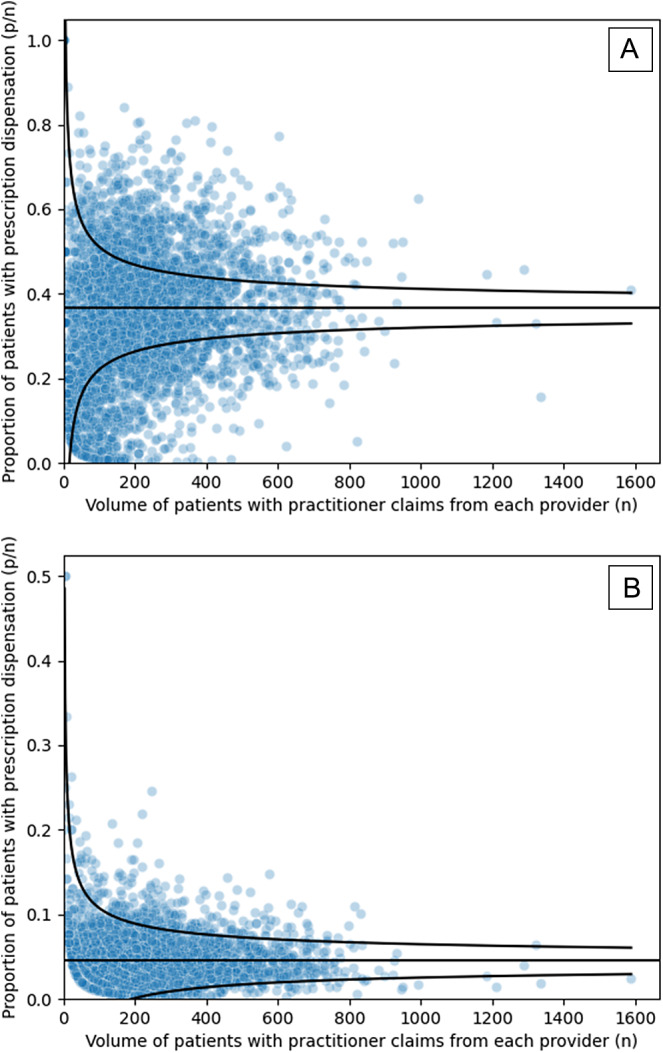
Funnel plots, showing high variation (A) in C10AA: HMG-CoA reductase inhibitors and low variation (B) in C03AA: Thiazides, plain. Both funnel plots are for prescribers primarily billing under the general practice specialty.

In the funnel plot showing high variation (Figure 2A), there were many prescribers falling outside of control limits relative to the number within control limits. In the funnel plot showing low variation (Figure 2B), a greater proportion of prescribers were within control limits. The number of prescribers likely to be affected reflects the number of data points outside of control limits. The number of patients likely to be affected was the product of the vertical distance of each prescriber from the control limits and the practice size, or the horizontal position. This outcome was strongly influenced by data points furthest from the control limits, particularly those with the largest practice sizes. The potential budgetary impact was also strongly influenced by the number of patients affected and the average price for drugs in the ATC class over the 2023 calendar year.

Twenty-one unique ATC classes were identified when ranked in descending order by the three outcomes considered above control limits (Table 2). There was much overlap between the number of patients and the number of prescribers likely to be affected. There was no overlap in the top ten ATC classes between potential budgetary impact and other outcomes. Five of the six ATC classes identified as having high-potential budgetary impact included biologic agents: L04AA, M05BX, L04AC, R03DX, and L04AB.

**Table 2.** Top ten ATC classes by outcome above control limits. Bold indicates the ATC class is in the top ten for the outcome column

ATC class	Specialty	Prescribers affected		Patients affected		Potential budgetary impact	
		Rank	Outcome	Rank	Outcome	Rank	Outcome
L04AA: Selective immunosuppressants, excluding corticosteroids	GAST	342	9	427	43	**1**	**$32,308,733**
	INMD	282	12	207	218	**2**	**$8,208,368**
	NEUR	919	2	572	21	**6**	**$1,878,247**
M05BX: Other drugs affecting bone structure and mineralization	INMD	380	8	282	117	**8**	**$1,752,044**
L04AC: Interleukin inhibitors	DERM	380	8	224	191	**3**	**$6,658,651**
R03DX: Other systemic drugs for obstructive airway diseases	RSMD	301	11	398	51	**4**	**$2,560,752**
N07XX: Other nervous system drugs	INMD	1374	1	365	66	**5**	**$2,009,959**
L04AB: Tumor necrosis factor alpha inhibitors	GAST	720	3	577	21	**7**	**$1,868,317**
	INMD	380	8	315	91	**10**	**$1,160,507**
A10BJ: Glucagon-like peptide–1 analogues	GP	51	218	48	1,883	**9**	**$1,373,055**
C10AA: HMG-CoA reductase inhibitors	GP	**1**	**794**	**1**	**20,276**	22	$433,414
G03CA: Natural and semisynthetic estrogens, plain	GP	**2**	**491**	17	4,114	21	$494,897
N06AX: Other antidepressants	GP	**3**	**461**	**3**	**7,466**	27	$328,390
A02BC: Proton pump inhibitors	GP	**4**	**459**	**4**	**6,828**	49	$157,163
M05BA: Bisphosphonates	GP	**5**	**452**	11	4,757	50	$149,574
H02AB: Glucocorticoids	GP	**6**	**440**	**6**	**5,951**	109	$50,798
H03AA: Thyroid hormones	GP	**7**	**431**	**5**	**6,502**	39	$225,770
C09CA: Angiotensin II receptor blockers, plain	GP	**8**	**431**	**2**	**7,485**	75	$90,226
C03CA: Sulfonamides, plain	GP	**9**	**394**	12	4,510	206	$16,392
C09AA: Angiotensin-converting enzyme inhibitors, plain	GP	**10**	**382**	15	4,273	77	$89,524
N02AA: Natural opium alkaloids	GP	13	367	**7**	**5,754**	19	$536,769
S01BA: Corticosteroids, plain	OPHT	141	37	**8**	**5,697**	62	$108,529
C08CA: Dihydropyridine derivatives	GP	12	368	**9**	**5,607**	64	$105,786
N02BE: Anilides	GP	22	318	**10**	**5,331**	198	$17,640

Abbreviations: ATC, anatomic therapeutic chemical; GAST, gastroenterology; INMD, internal medicine; NEUR, neurology; DERM, dermatology; RSMD, respiratory medicine; GP, general practice; OPHT, ophthalmology.

There was much overlap in the ATC class–prescriber specialty combinations identified above and below control limits (Tables 2 and 3). The magnitudes of outcomes below control limits were smaller than the magnitudes of outcomes above control limits. Similarly, outcomes of patients and prescribers affected were mostly for prescribers using “general practice” billing codes. Biologic agents identified as being below the control limits were less heavily represented due to their potential budgetary impact among ATC classes than those above control limits; however, five of ten combinations of ATC class and prescriber specialty still focused on biologic agents.

**Table 3.** Top ten ATC classes by outcome below control limits. Bold indicates the ATC class is in the top ten for the outcome column

ATC class	Specialty	Prescribers affected		Patients affected		Potential budgetary impact	
		Rank	Outcome	Rank	Outcome	Rank	Outcome
L04AA: Selective immunosuppressants, excluding corticosteroids	INMD	132	15	88	175	**1**	**$6,573,131**
	GAST	483	1	440	2	**2**	**$1,542,829**
L04AC: Interleukin inhibitors	DERM	195	8	183	42	**3**	**$1,471,217**
L04AB: Tumor necrosis factor alpha inhibitors	INMD	144	13	163	54	**4**	**$684,625**
	GAST	483	1	378	4	**9**	**$354,237**
N07XX: Other nervous system drugs	INMD	300	3	229	22	**5**	**$661,360**
M03AX: Other muscle relaxants, peripherally acting agents	PHMD	244	5	161	55	**6**	**$598,052**
S01LA: Ocular antineovascularization agents	OPHT	264	4	63	260	**7**	**$533,881**
C10AA: HMG-CoA reductase inhibitors	GP	**1**	**954**	**1**	**18,360**	**8**	**$392,447**
N05AX: Other antipsychotics	PSYC	229	6	160	56	**10**	**$323,845**
A02BC: Proton pump inhibitors	GP	**2**	**563**	**2**	**6,307**	18	$145,162
H03AA: Thyroid hormones	GP	**3**	**442**	**4**	**3,729**	38	$51,744
C09CA: Angiotensin II receptor blockers, plain	GP	**4**	**426**	**6**	**3,365**	22	$101,513
C09AA: Angiotensin-converting enzyme inhibitors, plain	GP	**5**	**406**	**5**	**3,388**	29	$70,996
C08CA: Dihydropyridine derivatives	GP	**6**	**400**	**7**	**3,001**	36	$56,627
N06AX: Other antidepressants	GP	**7**	**391**	**10**	**2,558**	20	$112,517
C07AB: Beta-blocking agents, selective	GP	**8**	**322**	12	2,150	68	$23,422
M05BA: Bisphosphonates	GP	**9**	**312**	18	1,119	50	$35,168
A10BA: Blood glucose-lowering drugs, excluding insulin	GP	**10**	**291**	15	1,632	88	$12,470
S01BA: Corticosteroids, plain	OPHT	50	53	**3**	**5,251**	24	$100,035
S01EE: Antiglaucoma preparations and miotics – prostaglandin analogues	OPHT	52	52	**8**	**2,943**	14	$227,490
S01ED: Antiglaucoma preparations and miotics, beta-blocking agents	OPHT	58	47	**9**	**2,900**	11	$303,584

Abbreviations: ATC, anatomic therapeutic chemical; INMD, internal medicine; GAST, gastroenterology; DERM, dermatology; PHMD, physical medicine and rehabilitation; OPHT, ophthalmology; GP, general practice; PSYC, psychiatry.

Twenty unique ATC classes were identified when ranked in descending order by at least one of the three outcomes considered below control limits (Table 3). There were considerable overlap between the outcomes of prescribers and patients affected and minimal overlap with potential budgetary implications. Only one ATC class, C10AA: HMG-CoA reductase inhibitors, was present in the top ten for all three outcomes. Active chemical substances in identified ATC classes are listed in the Supplementary Appendix.

## Discussion

We identified ATC class–prescriber specialty combinations for potential advancement through HTR processes. This work was guided by the process attributes of being data-driven, routine and replicable, actionable, enabling collaboration, and offering a high return on investment. To make this work data-driven, population-level administrative data formed the backbone of our novel method. We used the Python programming language, which can make replication straightforward and routine. We estimated three different outcomes for decision makers to assess the return on investment applicable to their perspective. ATC class–prescriber specialty combinations identified using this method could make an actionable focus for engagement and collaboration with those seeking to optimize prescription drug technology use.

ATC class–prescriber specialty combinations identified for the outcomes of prescribers and patients affected had much overlap, both above and below control limits. The identification of such ATC classes such as C10AA: HMG-CoA reductase inhibitors, N06AX: other antidepressants, A02BC: proton pump inhibitors, or C09AA: ACE inhibitors is consistent with their use in the treatment of highly prevalent conditions by general practitioners. Differences between the outcomes of patients affected and potential budgetary impact highlight the important role of price in identifying prescription drug targets for HTR. Biologic agents, which were heavily represented due to their high-potential budgetary impact, are often associated with higher prices than other agents on the ADBL.

Interestingly, the ATC class–prescriber specialty combinations offering the highest potential budgetary impact reflect drugs in the mid–upper price range systematically prescribed by relatively few prescribers, rather than the most dispensed or most expensive ATC classes. For example, the high-potential budgetary impact of “L04AA: Selective immunosuppressants, excluding corticosteroids” prescribed by internal medicine specialists reflects only nine prescribers above control limits.

The smaller magnitude of potential budgetary impact below control limits relative to above control limits within ATC class–prescriber specialty combinations likely highlights important differences between patients treated. The study population was limited to patients aged 65 years or greater, who are all eligible for the “Coverage for Seniors Program (27).” While cost-related concerns are an important consideration for access to prescription drugs, all patients who received care from included prescribers had this premium-free prescription drug insurance (23). An important next step will be examining the patients treated by these prescribers to identify systematic differences in other factors affecting prescription drug use, such as the burden of illness or the supply of specialists who might prescribe these agents.

Different outcomes presented here may be helpful depending on the decision maker’s perspective and the availability of policy levers to them. For the directors of a formulary, the potential budgetary impact may be the most useful in identifying prescription drug targets with the most significant impact on expenditures. An estimation of the number of prescribers affected may be helpful for organizations targeting change through other nonformulary mechanisms, such as academic detailing. Interventions seeking to change prescriber behavior could consider the number of prescribers targeted while maximizing the potential budgetary impact.

This work also represents a novel application of funnel plots. Single funnel plots are commonly used for comparing institutional performance and monitoring clinical behavior (22;28–31). Here, we generated funnel plots for many classes of prescription drugs simultaneously from the same data set and compared them to each other. We summarized variation in a metric measured in dollars to identify the processes where uncertainty in use has the greatest impact on the budget. Comparing funnel plots generated with a single, large data set adds to the utility of funnel plot methods for investigating variation.

The analytical approach used in this work is likely generalizable to other jurisdictions, where population-level prescription drug dispensation data are available and funnel plot analysis could be replicated. This analysis highlighted that the ATC class–prescriber specialty combinations identified as having the highest potential for reduction in budgetary impact were not those with the highest price or the greatest variation in dispensation rates. Both price and variation were important. This novel application of funnel plots could be replicated in other jurisdictions to generate context-specific estimates of outcomes beyond control limits and aid in the identification of candidate prescription drug technologies for HTR processes.

### Limitations

Firstly, this analysis assumes that each ATC class–prescriber specialty combination is a single process, where bringing prescriber practices toward the mean is desirable. It could be the case that within a single ATC class–prescriber specialty combination, such as “L04AA,” prescribed by internal medicine specialists, prescribers, or patient populations systematically differ. A practice focused on autoimmune conditions where many patients are treated with similar agents may not be comparable to a more general practice where patients are more heterogeneous. This may have resulted in increased numbers of data points outside of control limits than if prescriber and patient populations were more homogenous.

Secondly, the position of control limits at three standard deviations is unlikely to be clinically meaningful. In contrast to other methods of technology identification for HTR that begin with low-value care lists or surveillance of evidence (14), clinical evidence was not considered. Spiegelhalter (28) described methods to ensure that data points falling outside of control limits were correctly identified, including statistical adjustment to identify outliers, interval-based estimates of the mean, or stratification. Each of these methods requires additional information or acknowledgment of an acceptable level of variability, perhaps from clinical literature. Because we aimed to make comparisons across an entire formulary, gathering additional information was not feasible at scale. Acknowledging an acceptable level of variability would be as arbitrary as the conventionally accepted position of control limits at three standard deviations. Consistency of the approach across ATC classes and specialties is strength of the analysis, but the macroperspective adopted for this analysis reduces certainty in estimated outcomes for each ATC class.

Thirdly, our work was limited by the lack of cost data. Due to the confidential nature of discounts from list prices, cost data could not be obtained. The price paid by the ADBL (17) for each agent was used as a proxy for cost. However, this limited the ability to assess how costs experienced by the ADBL might be affected by a change in use. In a survey of institutional payers for prescription drugs from North America, Europe, and Australasia, Morgan et al. (32) described discounts commonly ranging from 20 to 60 percent of list price. Therefore, the relationship between potential budgetary impact and cost remains uncertain.

Fourth, the dose could not be considered at the scale of this study. The “defined daily dose” (DDD) is a commonly used metric in drug utilization studies, reflecting the assumed average maintenance dose for a drug for its main indication in adults (33). The Canadian Patented Medicine Prices Review Board (PMPRB) recommended that the DDD should not be used to interpret utilization, should not be used in cost analyses, and should not be applied in policy decisions (33). If single chemical substances were considered for funnel plots, DDD could have been used, despite PMPRB recommendations. While the DDD may be used to compare utilization of single agents between jurisdictions, there is no therapeutic equivalence between DDDs (33). Aggregation of chemical substances at the fourth level of the ATC system means that, even if the PMPRB recommended DDD use, this metric loses any meaning.

Because of the lack of clinical meaning of control limit position and price versus cost outcomes, we recommend that relative rankings of ATC class–prescriber specialty combinations for each outcome are more likely to be accurate than the exact magnitude of estimated outcomes.

### Future research

Additional work is required to explore the affected patients to make the identified ATC class–prescriber specialty combinations actionable for decision makers. For example, do patients vary in a way that reflects differences in the burden of illness or the geographic location of specialists who might prescribe these agents? Links to clinical evidence and collaboration with clinical experts will be required to understand if or where use might be optimized. Exploration of patient characteristics will give additional information to engage clinicians and decision makers and identify where variation is problematic and actionable.

## Conclusions

We used a novel method comparing funnel plots to identify ATC class–prescriber specialty combinations with marked variation in use. Depending on the perspective and priorities of those undertaking HTR of prescription drugs and the available policy levers, different outcomes may be helpful in technology identification. This analysis is a starting point, and further efforts should focus on exploring the affected patients and making findings actionable for decision makers.

## Supporting information

Hofmeister et al. supplementary material Hofmeister et al. supplementary material

## Supplementary material

The supplementary material for this article can be found at http://doi.org/10.1017/S026646232510322X.
