# Supplementary material for: A method to identify prescription drug targets for health technology reassessment
Source: Int J Technol Assess Health Care. 2025 Nov 28;41(1):e81. doi: 10.1017/S026646232510322X (PMC12689240; doi:10.1017/S026646232510322X)
Supplement: Hofmeister et al. supplementary material [file S026646232510322Xsup001.docx]

Supplementary File 1. Active chemical substance(s) in ATC classes identified

| **ATC Class** | **Description** | **Included Drugs** |
| --- | --- | --- |
| C10AA | HMG CoA reductase inhibitors | Rosuvastatin, atorvastatin, simvastatin, pravastatin, lovastatin, fluvastatin |
| G03CA | Natural and semisynthetic estrogens, plain | Estradiol, conjugated estrogens |
| N06AX | Other antidepressants | Trazodone, mirtazapine, duloxetine, venlafaxine, bupropion, vortioxetine, tryptophan |
| A02BC | Proton pump inhibitors | Pantoprazole, omeprazole, lansoprazole, rabeprazole |
| M05BA | Bisphosphonates | Alendronic acid, risedronic acid, zoledronic acid, clodronic acid, pamidronic acid |
| H02AB | Glucocorticoids | Prednisone, dexamethasone, Trimacinolone, methylprednisolone, hydrocortisone, cortisone, betamethasone, prednisolone |
| H03AA | Thyroid hormones | Levothyroxine sodium, thyroid gland preparations, liothyronine sodium |
| C09CA | Angiotensin II receptor blockers, plain | Candesartan, irbesartan, telmisartan, valsartan, olmesartan medoxomil, losartan, eprosartan |
| C03CA | Sulfonamides, plain | Furosemide |
| C09AA | Angiotensin-converting enzyme inhibitors, plain | Perindopril, ramipril, lisinopril, enalapril, trandalopril, fosinopril, cilazapril, quinapril, captopril, benazepril |
| N02AA | Natural opium alkaloids | Hydromorphone, oxycodone, morphine, codeine combinations with psycholeptics |
| C07AB | Beta blocking agents, selective | Metoprolol, bisoprolol, atenolol, acebutolol |
| S01BA | Corticosteroids, plain | Prednisolone, fluorometholone, dexamethasone |
| C08CA | Dihydropyridine derivatives | Amlodipine, nifedipine, felodipine |
| N02BE | Anilides | Paracetamol |
| L04AA | Selective immunosuppressants, excluding corticosteroids | Leflunomide, upadacitinib, abatacept, vedolizumab, tofacitinib, baricitinib, eculizumab, fingolimod, teriflunomide, ocrelizumab, Siponimod, ofatumumab, cladribine |
| M05BX | Other drugs affecting bone structure and mineralization | Denosumab, burosumab |
| L04AC | Interleukin inhibitors | Ustekinumab, tocilizumab, secukinumab, ixekizumab, risankizumab, sarilumab, bimekizumab, tildrakizumab, anakinra |
| R03DX | Other systemic drugs for obstructive airway diseases | Mepolizumab, omalizumab, benralizumab |
| N07XX | Other nervous system drugs | Tafamidis, tetrabenazine, riluzole, patisiran, edaravone, inotersen, amifampridine |
| L04AB | Tumor necrosis factor alpha inhibitors | Adalimumab, etanercept, golimumab, infliximab, certolizumab pegol |
| A10BJ | Glucagon-like peptide-1 analogues | Semaglutide, lixisenatide |
| A10BA | Blood glucose lowering drugs, excluding insulins | Metformin |
| S01EE | Antiglaucoma preparations and miotics – prostaglandin analogues | Latanoprost, bimatoprost, latanoprostene bunod, travoprost |
| S01ED | Antiglaucoma preparations and miotics, beta blocking agents | Timolol combinations, timolol, betaxolol |
| M03AX | Other muscle relaxants, peripherally acting agents | Botulinum toxin |
| S01LA | Ocular antineovascularisation agents | Aflibercept, ranibizumab, brolucizumab |
| N05AX | Other antipsychotics | Risperidone, aripiprazole, brexpiprazole, paliperidone |
